# Medical and Philosophical News

**Published:** 1781-02

**Authors:** 


The Academy of Sciences at Bourdeaux having offered a prize of 450 livres for the beſt paper on the incontinence of urine, to which many perſons are ſubject when in bed, and on the means of curing it, M. Leger, a ſurgeon at Paris, has, without any view to the prize, communicated to the public a remedy, by means of which he has ſucceeded in three caſes of this ſort. It conſiſts of ſix grains of cantharides in powder, mixed with two drachms of extract of borage, and divided into 24 doſes, one of which is to be taken every night at bed-time. In one of the patients, a young lady, 24 years old, who had been ſubject to the above-mentioned complaint from her infancy, the medicine was repeated 72 nights, and the doſe of cantharides increaſed a few grains. This patient was perf[illegible]ly cured, and for two years has enjoyed the beſt health. The two other patients were ſiſters, the eldeſt fifteen and the youngeſt thirteen years old. The remedy produced in both the ſame good effect as in the caſe juſt now mentioned, and they have now been well a year. By way of precaution, they were all directed to drink linſeed tea, but the medicine occaſioned no ſtrangury in either of them.

